# Quantum entanglement dynamics of spacetime and matter

**DOI:** 10.1016/j.fmre.2023.10.004

**Published:** 2023-10-29

**Authors:** Zeng-Bing Chen

**Affiliations:** National Laboratory of Solid State Microstructures and School of Physics, Nanjing University, Nanjing 210093, China

**Keywords:** Quantum gravity, Spacetime-matter entanglement, Information completeness, Quantum entanglement dynamics, Trinity

## Abstract

It was known long ago that quantum theory and general relativity are in sharp conflict in their foundations. Their fundamental inconsistencies render a consistent theory of quantum gravity the most challenging problem in physics. Here we propose an information-complete quantum field theory (ICQFT), which describes elementary fermions, their gauge fields, and gravity (together, called the trinary fields) as an elementary trinity without any conceptual inconsistency of existing theories. The ICQFT unifies matter and spacetime (gravity) as information via spacetime-matter entanglement, which encodes complete physical predictions of the theory and leads to a compelling solution to the problem of time. We consider two particular forms of spacetime-matter entangled states and their physical consequences. One of them results in a universal relation between entanglement entropy and geometry (area and volume), allowing us to determine the cosmological constant term in the classical Einstein equation. Based on a quantum-information definition of dark energy, our Universe is not strictly holographic. We predict the interior quantum state of a Schwarzschild black hole to be maximally information-complete. As a concrete quantum formulation of gravity coupled with matter, the ICQFT is quantum entanglement dynamics for spacetime and matter and eliminates the conceptual obstacles of existing quantum gravity theory.

## Graphical abstract

**Figure:**
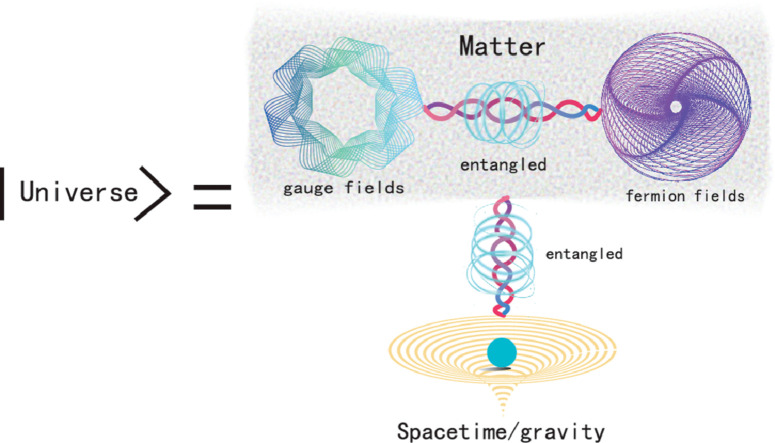

## 1 Introduction

Quantum theory—quantum mechanics and quantum field theory (QFT)—and general relativity are two pillars of our current physics and deeply impact even our daily life. The achievements motivated by either of the two pillars are remarkable. However, it was recognized long ago that quantum theory and general relativity are in sharp conflict in their foundations as summarized by Thiemann in a beautiful review [1]. Einstein’s equation relates the geometry of spacetime and the energy-momentum tensor of matter. In Thiemann’s terminology, on one hand, the classical-quantum inconsistency means that, while the matter fields are well described by the Standard Model in flat spacetime, the geometry of spacetime is described by the classical Einstein equation. On the other hand, general relativity results in the unavoidable existence of spacetime singularities, where all laws of physics are doomed to fail. Such an instability of spacetime and matter implies the internal inconsistency of general relativity. At the same time, conventional QFT suffers from the notorious infrared and ultraviolet singularities (divergences). Although the divergences can be “get rid of” by renormalization as in the Standard Model, renormalization fails when it applies to general relativity.

The fundamental inconsistencies mentioned above motivate a long march of quantizing gravity—“quantum theory’s last challenge” [2]. Among various existing approaches to quantum gravity, loop quantum gravity [1], [3], [4], [5], [6], [7], [8], [9] is very impressive for, among others, its prediction of discrete structure of spacetime and the entropy counting of black holes. However, almost all, if not all, existing methods to quantum gravity tacitly assume, explicitly or implicitly, the completeness of conventional quantum theory. Logically, it is totally possible that the fundamental inconsistencies of our current theories could be caused by the incompleteness of quantum theory. The debate on the real meaning of quantum states [10], [11], [12] and on the quantum measurement problem [13], [14], [15], [16] occupies the whole history of quantum theory. Notice that conventional QFT in curved spacetime has its own interpretational problems, e.g., the black-hole information paradox [17] and the physical meaning on the usual concept of particles [18], [19], [20]. These interpretational difficulties of quantum theory motivate various interpretations [21], or understanding quantum theory from different angles [22], [23], [24].

Yet, these interpretations or fresh understanding seldom challenges the completeness of quantum theory. The most serious challenge stems from the famous Einstein-Podolsky-Rosen paper [10] questioning the completeness of current quantum description against local realism. The follow-up discover of Bell’s inequalities [25] and their various experimental tests give us an impression that quantum mechanics wins against the Einstein-Podolsky-Rosen argument. The interpretation on violations of Bell’s inequalities as quantum nonlocality was questioned from the many-worlds picture [26].

Recently, we took a totally different way of thinking. We suggested an information-complete quantum theory (ICQT) by assuming that quantum states represent an information-complete code of any possible information that one might access to a physical system [27]. The key to this development is the information-completeness in the trinary picture. A single, free physical system in our conventional sense is excluded from the outset by the ICQT as it is simply meaningless for acquiring information, which must be accessed via interaction (entanglement). The two-party (a physical system S plus its measurement apparatus A) picture as used in current quantum mechanics was argued to be information-incomplete. To fulfill the information-completeness such that any information must be carried or acquired by certain quantum system, one has to adapt a trinary description, in which the third system, called the “programming” system (system P), has to be introduced. Then the whole system P−SA (the “trinity”) possesses a particular self-defining quantum structure without the usual measurement postulate. But in the context of conventional quantum mechanics, one could introduce more programming systems $P^{\prime }$, $P^{\prime \prime }$... to programe P−SA, $P^{\prime }\;-\;\left(P\;-\;\mathrm{SA}\right)$..., known in the usual quantum measurement model as the von Neumann chain, which is mathematically unlimited. As we argued previously, if P is spacetime being a physical quantum system, the von Neumann chain is terminated as there is no spacetime beyond spacetime. Now we immediately see that, to arrive at an information-complete and self-defining quantum structure, spacetime must be a quantized physical system, as well as the programming system. Meanwhile, general relativity tells us that spacetime is indeed a physical system and the same thing as gravity.

Now a fascinating thing happens here. On one hand, a genuine ICQT requires that spacetime/gravity must be quantized and plays a very specific role in its own formulation. On the other hand, we do have the most remarkable trinity of nature—matter fermions, their gauge fields, and gravity (spacetime); the role of the Higgs field will be considered elsewhere [28]. In the present work, we generalize the idea of the ICQT and present an information-complete QFT (ICQFT), which describes elementary fermions, their gauge fields, and gravity as an indivisible quantum trinity. By its very construction, the ICQFT provides a coherent picture and conceptual framework of unifying matter and spacetime (gravity) as information via spacetime-matter entanglement. Such a quantum information (or, entanglement) dynamics of spacetime and matter represents thus a candidate unifying quantum theory and general gravity into a single, consistent theory. The formulation gives up the probability description of current quantum mechanics and does not need the vague concepts such as observers and wave-function collapse. The theory describes a self-defining or self-explaining Universe that is genuinely quantum; there is no room for any classical systems or concepts. We consider some applications of the ICQFT. First, we give a compelling solution to the problem of time, well-known in quantum gravity community, and a quantum information definition of dark energy. Second, a particular form of spacetime-matter entanglement allows us to give a correct classical limit (i.e., the classical Einstein equation). Then we conjecture a spacetime-matter state of the Universe, permiting a natural generalization of the holographic relation, in which an extra term is argued to be related to dark energy and the cosmological constant term in Einstein’s equation. Finally, we predict the interior quantum state of a Schwarzschild black hole to be maximally information-complete. Central to our development is the idea of spacetime-matter entanglement that encodes complete physical predictions of the theory. In our formalism, entanglement is thus universal just like that gravity is universal; the universal entanglement is the glue of spacetime and matter and thus the building block of the world.

## 2 Information-complete quantum fields and the state-dynamics postulate

**Fig. 1:**
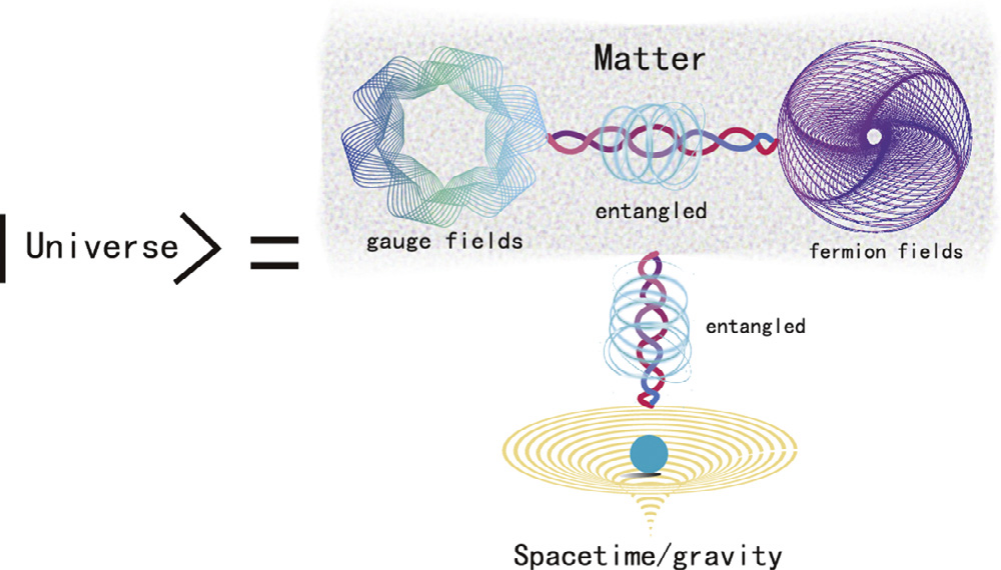
**The fundamental trinity of nature**. Here spacetime-matter entanglement “glues” spacetime and matter (matter fermions and their gauge fields) as an indivisible trinity, encodes information-complete physical predictions of the world, and is as universal as universal gravitation.

In the present case of the ICQFT, an elementary fermion (e.g., a Dirac electron) field and its corresponding gauge field are called as system S and system A, respectively; SA together as matter fields. The gravitational field (i.e., quantized spacetime) is the programming system (system P). The trinary description (see Fig. 1) then corresponds to a dual entanglement pattern among the three systems: The matter fields (S and A together) and gravity are mutually defined by interacting and entangling each other; the fermion field (S) and the gauge field (A), both programmed by gravity, are likewise entangled and mutually defined. Here spacetime plays a role of quantum memory that stores or encodes all entanglement patterns for the fermion field and its gauge field. Thus, in the ICQFT the viewpoint on spacetime and matter is dramatically different from our previous picture. Neither spacetime nor matter is an isolated entity; they must be described as a trinity and entangled in the dual form to make sense for acquiring information. In this sense, the ICQFT is quantum entanglement dynamics of the trinity, in which quantized spacetime plays a unique role for formulating the theory.

To illustrate the basic idea, for concreteness we only consider the gravitational field interacting with two kinds of matter fields: the fermion field $\hat{\psi }\left(x\right)=\hat{\psi }\left(x,t\right)$ of a Dirac particle (with mass m and charge q) and the electromagnetic field ${\hat{F}}_{\mu \nu }=\partial_{\mu }{\hat{A}}_{\nu }-\partial_{\nu }{\hat{A}}_{\mu }$, where ${\hat{A}}_{\mu }$ is the electromagnetic potential vector. The generalization to non-Abelian gauge fields is straightforward. Here we adapt notions as used in Rovelli’s book [7]. A spacetime coordinate $x=(x^{\mu })$ with μ,ν,…=0,1,2,3 being spacetime tangent indices. The gravity is described by the tetrad field $e_{\mu }^{I}\left(x\right)$, which relates to the usual metric tensor $g_{\mu \nu }$ by $g_{\mu \nu }\left(x\right)=\eta_{IJ}e_{\mu }^{I}\left(x\right)e_{\nu }^{J}\left(x\right)$. Indices I,J,… label the Minkowski vectors and the Minkowski metric $\eta_{IJ}$ has signature [−,+,+,+]. The total action of the trinary fields is $S\left(\hat{e},\hat{\omega };\hat{A},\hat{\psi }\right)=S_{G}\left(\hat{e},\hat{\omega }\right)+S_{M-G}\left(\hat{e},\hat{\omega };\hat{A},\hat{\psi }\right)$, where $S_{M}\left(\hat{e},\hat{\omega };\hat{A},\hat{\psi }\right)=S_{\mathrm{Dirac}}\left(\hat{e},\hat{\omega };\hat{\psi },\hat{A}\right)+S_{\mathrm{EM}}\left(\hat{e};\hat{A}\right)$. Here we only write down explicitly the action for Dirac’s field to introduce notations:

(1) $$S_{\mathrm{Dirac}}=\frac{1}{2}\int dx^{4}\hat{e}\bar{\psi }\left[\gamma^{I}{\hat{e}}_{I}^{\mu }iD_{\mu }-m\right]\hat{\psi }+H.C.$$

where $\bar{\psi }={\hat{\psi }}^{†}\gamma^{0}$, $D_{\mu }=\partial_{\mu }+{\hat{\omega }}_{\mu J}^{I}L_{\;I}^{J}-iq{\hat{A}}_{\mu }$, $\hat{e}$ is the determinant of ${\hat{e}}_{I}^{\mu }$, γ’s are the Dirac matrices, $\hat{\omega }$ is the spin connection, and $L_{\;I}^{J}$ are the generators of the Lorentz algebra.

Due to the presence of quantized spacetime, it is convenient to work in the Hamiltonian formalism [7], [8], [9], which is better established in loop quantum gravity. There, the dynamical variables, in terms of ${\hat{e}}_{I}^{\mu }$ and ${\hat{\omega }}_{\mu J}^{I}$, are the Ashtekar-Barbero [29], [30] connection ${\hat{B}}_{a}^{i}\left(\tau \right)$ [defined on a three-dimensional surface without boundaries; a,b,…=1,2,3 are spatial indices and i,j,…=1,2,3 take values in the Lie algebra su(2)] and the “gravitational electric field” ${\hat{E}}_{b}^{j}\left(\tau \right)$, which is the i8πγG (γ: the Immirzi parameter [31]) times momentum conjugate to ${\hat{B}}_{a}^{i}\left(\tau \right)$.

According to the ICQT and as we pointed out above, we must describe the trinity ($\hat{\psi }$, $\hat{A}$, and $\hat{B}$) as a single, information-complete physical system. We can formally write the entangled state of the trinity as |B,ψ,A〉, which always allows a P−AS Schmidt decomposition with two orthogonal bases of the matter field ($\hat{\psi },\hat{A}$) sector and the gravitational field ($\hat{B}$) sector. This spacetime-matter entanglement in the Schmidt form encodes complete information of the trinity such that matter and spacetime are mutually defined. As programmed by each state of gravity in the P−AS Schmidt decomposition, the $\hat{\psi }\;-\;\hat{A}$ entanglement encodes complete information of the matter field sector such that the fermion field $\hat{\psi }$ and the gauge field $\hat{A}$ are mutually defined.

Thus, in the present field-theoretical case, the trinity is also entangled in dual form [27], to be given explicitly below. Here only pure-state entanglement appears in our description and is uniquely quantified by the usual entanglement entropy [32], [33]. The property of the pure-state entanglement entropy indicates that, while states for each field is relative, their information encoded in dual entanglement is invariant under the changes of “local” (single-field) bases, i.e., under any unitary transformations upon states of a single field. This is in a perfect analog to the spirit of general relativity, in which physical observables must be invariant under general coordinate transformations. Now let us state the key point of the ICQFT—*quantum relationalism*: Complete information (namely, all physical predictions) of the trinary fields (fermions, their gauge fields, and gravity) is encoded in dual entanglement; fields involved in the dual-entanglement structure should be mutually defined, as we specified above, to obey the information-completeness.

According to our previous definition of information-complete physical systems [27], information-complete field states and field operators can only be defined in the Hilbert spaces of the matter field ($\hat{\psi },\hat{A}$) sector and the gravitational field ($\hat{B}$) sector. The $\hat{\psi }\;-\;\hat{A}$ entanglement, programmed by each state in the orthogonal basis of the gravitational field sector, encodes information for information-incomplete field in the Hilbert space of $\hat{\psi }$ or $\hat{A}$. Thus, the ICQFT describes nature with the basic trinity that is in a specific entanglement structure. The physical significance of our current understanding on matter fermion fields and gauge fields is completely changed in the ICQFT: Either a fermion field or its gauge field alone loses its physical significance and cannot be regarded as isolated, physical (information-complete) entities; only jointly they define spacetime and can be described as an information-complete physical entity. This immediately explains, to be shown below, the occurrence of the black-hole information paradox [17] as an unavoidable consequence of the conventional information-incomplete description.

What is the dynamics leading to the proposed entanglement structure of the whole system? If we include all gauge and fermion fields into the total action $S(\hat{B};\hat{A}\ldots ,\hat{\psi }\ldots )$, then the ICQFT, by definition, is a theory about the whole Universe. All predictions of the theory have to be made without the assumption of externally given observers [27] and initial/boundary conditions, thus excluding the applicability of existing approaches such as Schwinger’s action principle and Feynman’s path integral. Nevertheless, the two mentioned approaches motivate us to suppose, as a basic postulate (“the state-dynamics postulate”) of the ICQFT, that the Universe is self-created from no spacetime and no matter with the least action (ℏ=1):

(2) $$\begin{array}{cc} & |B;A\ldots ,\psi \ldots \rangle =e^{iS(\hat{B};\hat{A}\ldots ,\hat{\psi }\ldots )}|\emptyset \rangle \\ & \delta S\left(\hat{B};\hat{A}\ldots ,\hat{\psi }\ldots \right)|B;A\ldots ,\psi \ldots \rangle =0 \end{array}$$

Here $|\emptyset \rangle \equiv |\emptyset_{G}\rangle ⊗|\emptyset_{M}\rangle$ is the common empty state of matter (the empty-matter $|\emptyset_{M}\rangle$) and geometry (the empty-geometry state $|\emptyset_{G}\rangle$ in loop quantum gravity [7]). Note that the requirement of the least action gives the constraint conditions and the equations of motion as usual, but with an interesting new feature, namely, the kinematics and dynamics of the theory is indivisible. To see the feature, note that the dynamical law and states always appear jointly in the postulate (Eq. 2). This is in sharp contrast to the tradition where the dynamical law [i.e., $\delta S(\hat{B};\hat{A}\ldots ,\hat{\psi }\ldots )=0$] and states are given separately. Thus, quantum entanglement dynamics of the trinary fields under study unifies the dynamical law and states, a feature required by the information-complete trinary description.

To summarize, the information-completeness principle puts a profound restriction on what are physical systems and how to describe the physical systems. Quantum relationalism stated above gives precisely an information-interpretation of the gauge invariance in conventional QFT under local Lorentz transformations, local gauge transformations, and diffeomorphism [7]. Namely, given the conventional gauge invariance, complete information of the trinary fields is invariant. However, it seems that the information completeness puts stronger restrictions on our field-theoretical description than the usual gauge invariance; for instance, either $\hat{\psi }$ or $\hat{A}$ alone is information-incomplete field in the ICQFT. To be more clear on this point, let us recall that a harmonic oscillator in classical mechanics can have any continuous positive energy. But quantum mechanics restricts its energy to be discrete values such that the oscillator’s classical state space that is previously physical according to classical principles is now severely constrained by quantum principles. Here the situation is completely the same. The information-completeness principle, or put differently, the kinematics-dynamics indivisibility of the theory, severely restricts the allowed state space of the quantized fields such that the originally physical states in conventional QFT become unphysical in our new description. Below let us show how the ICQFT leads to a conceptually compelling picture of unifying gravity and matter.

## 3 Quantum state of gravity and matter

The classical Einstein field equation reads [7]

(3) $$R_{\mu }^{I}-\frac{1}{2}\left(R-2\Lambda \right)e_{\mu }^{I}=8\pi GT_{\mu }^{I}$$

where $R_{\mu }^{I}$ (R) is the Ricci tensor (scalar), $T_{\mu }^{I}$ the energy-momentum tensor of matter; Λ (G) represents the cosmological (Newton) constant. In classical domains, Einstein’s equation is extremely successful. But quantum mechanically, it looks problematic as one has to quantize these fields therein. As argued by Thiemann [1], in quantum gravity theory $T_{\mu }^{I}$ should be quantized to be a field operator ${\hat{T}}_{\mu }^{I}\left(\hat{e},\hat{\omega }\right)$ in the Hilbert space of both spacetime and matter. However, the problem (called hereafter as the “Hilbert-space inconsistency”, which also applies to usual interacting quantized fields) still exists: Both sides of Einstein’s equation belong to different Hilbert spaces as the left are purely operators for spacetime geometry; generally there is no way of equating them. To remedy the inconsistency, one could of course act both sides of the quantized Einstein equation upon a joint state of spacetime and matter such that the equality for field operators is mapped into the equality for classical field variables as in Eq. 3; see also the second line of Eq. 2. Then another problem arises as to what the joint state of spacetime and matter is. As we will show below, the ICQFT provides a concrete way to find the appropriate joint state of spacetime and matter to “glue” the two pieces of Einstein’s equation.

Conceptual inconsistencies and difficulties of formulating a concrete quantum gravity theory motivates the idea that Einstein’s equation is merely an effective spacetime theory [34], [35]; it cannot be quantized at all in a way that we quantize matter fields. Thanks to the development of loop quantum gravity, some conceptual inconsistencies and difficulties of the quantum gravity theory have been overcome.

For the trinary fields under study, we need to specify a conservative quantity (commutative with the gravitational Hamiltonian and with the matter Hamiltonian) as a programming observable [27]. As we noted in the context of the ICQT, the choice of the programming observable is relative so that we could use gravity to programme matter, or vice versa. As the matter field couples to gravity via its energy-momentum tensor (keeping in mind that conservation of the energy-momentum tensor in curved spacetime is a subtle issue), it seems to be natural to programme gravity by matter. However, as gravity couples universally to all matter species, it is conceptually compelling to programme matter by gravity. In this case, the Hamiltonian of the trinary fields can then be formally written as

(4) $$\begin{array}{ccc} H_{G+M} & = & \int d^{3}xH_{M\text{+}G}\left(\hat{\psi },\hat{A};{\hat{B}}_{a}^{i},{\hat{E}}_{b}^{j}\right)\\ & = & \int d^{3}xH_{G}\left({\hat{B}}_{a}^{i},{\hat{E}}_{b}^{j}\right)+\int d^{3}xH_{M\text{-}G}\left(\hat{\psi },\hat{A};{\hat{B}}_{a}^{i},{\hat{E}}_{b}^{j}\right)\\ & = & H_{G}\left({\hat{B}}_{a}^{i},{\hat{E}}_{b}^{j}\right)+H_{M\text{-}G}\left(\hat{\psi },\hat{A};{\hat{B}}_{a}^{i},{\hat{E}}_{b}^{j}\right) \end{array}$$

where H denotes the Hamiltonian density ($H_{G}$ for gravity, $H_{M\text{+}G}$ for gravity and matter, and $H_{M\text{-}G}$ for the gravity-matter coupling); $\bar{\psi }$ and momentum conjugate to $\hat{A}$ are all omitted for notation simplicity.

Although the matter-field sector of the problem is less developed [7], [8], [9], the gravity sector is well established within loop quantum gravity so that, with the input of the information-completeness principle, we can write the dual spacetime-matter entangled state, resulting from the state-dynamics postulate, in the standard Schmidt form as

(5) $$|B,(\psi ,A)\rangle =\sum_{s}S_{G+M}\left[s\right]|B,s\rangle ⊗|(\psi ,A),s\rangle$$

here $S_{G+M}\left[s\right]$ (>0) denotes the Schmidt coefficients and is determined by dynamics of the trinary system; {|B,s〉} (named as the “programming basis” in the ICQT) and {|(ψ,A),s〉} span orthogonal bases for the Hilbert spaces of spacetime and matter, respectively. Without loss of generality we assume s to be discrete. In loop quantum gravity we do have the spin-network basis of discrete spectra [7], [9]. With respect to this specific decomposition, the programmed entangled state |(ψ,A),s〉 for the Dirac field and the electromagnetic field can be likewise decomposed as

(6) $$|(\psi ,A),s\rangle =\sum_{\ell }S_{M|G}\left[\ell ,s\right]|A,\ell ,s\rangle ⊗|\psi ,\ell ,s\rangle$$

which encodes complete information about $\hat{\psi }$ and ${\hat{A}}_{\mu }$ as programmed by |B,s〉. {|ψ,ℓ,s〉} and {|A,ℓ,s〉} are two orthogonal bases for the Hilbert spaces of the Dirac field and the electromagnetic field.

Let us consider the physical significance of dual entanglement in Eqs. 5–6. Dual entanglement in Eqs. 5–6 already implies the existence of discrete orthonormal bases {|B,s〉}, {|(ψ,A),s〉}, {|ψ,ℓ,s〉}, and {|A,ℓ,s〉}, as a direct consequence of our information-complete description. The information-completeness in turn implies that all these states [|B,s〉, |(ψ,A),s〉, |ψ,ℓ,s〉, and |A,ℓ,s〉] involved in the dual Schmidt forms have to be physical states in their corresponding state spaces, eliminating any gauge arbitrariness, of the trinary system as they encode all the relevant information of direct *physical* significance (namely, physical predictions of the theory).

One of the most important results achieved by loop quantum gravity is the identification of the “spin-network” states as the Hilbert space of quantized gravity. These states are diffeomorphism-invariant, form a discrete orthonormal basis, and support discrete spacetime geometry [1], [3], [4], [5], [6], [7], [8], [9]. Now an important problem arises: What is the relation between the spin-network states and |B,s〉 in Eq. 5? Below we will give first of all a rigorous (but formal) formulation of the ICQFT. Then we make a conjecture, in which the spin-network states span the programming basis.

## 4 Dual dynamics and solution to problem of time

As constraint physical systems, the trinary fields satisfy the Gauss, the diffeomorphism, and the Hamiltonian constraints which must annihilate the physical state [7], [8], [9], e.g.

(7) $$H_{G+M}|B,(\psi ,A)\rangle =0$$

for the Hamiltonian constraint. As a result of Eq. 7, the whole system seems to have no time evolution, a fact known as the “problem of time” [7], [9], [36] in quantum gravity and quantum cosmology. As we noted above, for our purpose we need a programming observable, commutative with the gravitational Hamiltonian $H_{G}\left({\hat{B}}_{a}^{i},{\hat{E}}_{b}^{j}\right)$ and the total Hamiltonian $H_{G+M}$. Now let us show how we can obtain the observable.

The spacetime-matter state |B,(ψ,A)〉 can of course be expanded in any orthogonal bases for the Hilbert spaces of gravity and matter. We thus can freely choose a basis such that the reduced density operator for gravity has only positive diagonal elements, namely

(8) $$\begin{array}{cc} \rho_{G}^{\left\{s\right\}} & ={\mathrm{tr}}_{M}\left[|B,(\psi ,A)\rangle \langle B,(\psi ,A)|\right]\\ & =\sum_{s}S_{G+M}^{2}\left[s\right]|B,s\rangle \langle B,s| \end{array}$$

where ${\mathrm{tr}}_{M}$ means trace over the matter state space. Note that this diagonal form of $\rho_{G}^{\left\{s\right\}}$ can always be achieved by a unitary transformation upon the Hilbert space *merely for gravity*. We can also rewrite Eq. 8 as

(9) $$\rho_{G}^{\left\{s\right\}}\equiv \frac{e^{-{\hat{I}}_{\mathrm{Eu}}}}{\mathrm{tr}(e^{-{\hat{I}}_{\mathrm{Eu}}})}\equiv \frac{e^{-\beta \hat{\Xi }}}{\mathrm{tr}(e^{-\beta \hat{\Xi }})}$$

where $\beta =1/(\kappa_{B}T)$ with $\kappa_{B}$ being the Boltzmann constant and T having dimension of temperature; a Hermitian operator $\hat{\Xi }$, which is positive semidefinite, is named as the “entanglement Hamiltonian” in other contexts [37], [38]. Hereafter we call ${\hat{I}}_{\mathrm{Eu}}$ the *Euclidean entanglement action*, whose spectrum contains complete information of $\rho_{G}^{\left\{s\right\}}$ and thus, spacetime-matter (but not matter-matter) entanglement. We conjecture that ${\hat{I}}_{\mathrm{Eu}}$ might be given by certain Euclidean action of gravity. We leave this conjecture for future work; for the Euclidean action of gravity, see, e.g., Refs. [39], [40].

The information-completeness within a trinary description demands that

(10) $$\begin{array}{cc} H_{G+M} & =H_{G}+H_{M\text{-}G}\\ H_{M\text{-}G} & =\sum_{s}|B,s\rangle \langle B,s|⊗H_{M|G}\left(\hat{\psi },\hat{A};s\right) \end{array}$$

where gravity and matter are coupled with $H_{M\text{-}G}$ of a factorizable form, and $H_{M|G}\left(\hat{\psi },\hat{A};s\right)$ is the matter Hamiltonian conditional on (namely, programmed by) the gravity state |B,s〉. The overall evolution of the gravity-matter system is given in Heisenberg’s picture by

(11) $$\begin{array}{cc} |B,(\psi ,A)\rangle & ={\hat{U}}_{G+M}\left(t\right)|\emptyset \rangle \\ {\hat{U}}_{G+M}\left(t\right) & =\sum_{s}|B,s\rangle \langle B,s|{\hat{U}}_{G}\left(t\right)⊗{\hat{U}}_{M|G}\left(s,t\right) \end{array}$$

The evolution operator ${\hat{U}}_{G+M}$ also has a factorizable structure and is determined by

(12) $$\begin{array}{cc} i\frac{\partial }{\partial t}{\hat{U}}_{G}\left(t\right) & =H_{G}{\hat{U}}_{G}\left(t\right)\\ i\frac{\partial }{\partial t}{\hat{U}}_{M|G}\left(s,t\right) & =H_{M|G}{\hat{U}}_{M|G}\left(s,t\right) \end{array}$$

As we have chosen a particular basis for gravity as in Eq. 8, the basic property of the Schmidt decomposition [41] leads to that all $|(\psi ,A),s;t\rangle \equiv {\hat{U}}_{M|G}\left(s,t\right)|\emptyset_{M}\rangle$ form an orthonormal basis and

(13) $${\hat{U}}_{G}\left(t\right)|\emptyset_{G}\rangle =\sum_{s}S_{G+M}\left[s,t\right]|B,s\rangle$$

The dynamical evolutions in Eqs. 11 and 12 take the desired form as in the ICQT. They are mutually defined for spacetime and matter as expected. A similar dynamics can be obtained for the matter fermion field and the gauge field. The dual dynamical evolution always results in correct dual entanglement, in which all constituent states are ensured to be physical. Now it is ready to see that dual dynamics is a robust feature of our information-complete trinary description. As can be seen from Eq. 7, using Eqs. 11 and 12 yields

(14) $$\frac{\partial }{\partial t}{\hat{U}}_{G+M}\left(t\right)=0$$

namely, the whole system (spacetime+matter) cannot have a dynamical evolution, indeed. Yet, both spacetime and matter have their own dynamical evolutions, which are “glued” by spacetime-matter entanglement. Thus, the problem of time, remaining as one of the conceptual obstacles for a consistent quantum gravity, disappears in our formalism.

Eq. 13 and the factorizable form of ${\hat{U}}_{G+M}\left(t\right)$ have a physically appealing interpretation as follows. ${\hat{U}}_{G}\left(t\right)$, exactly like a quantum gate (the “gravity gate”), prepares the gravity state $\sum_{s}S_{G+M}\left[s,t\right]|B,s\rangle$ as the controlling state from $|\emptyset_{G}\rangle$. Then the controlled-${\hat{U}}_{M|G}$ operation (the “gravity-matter gate”) $\sum_{s}|B,s\rangle \langle B,s|⊗{\hat{U}}_{M|G}\left(s,t\right)$ creates the gravity-matter entangled state |B,(ψ,A)〉. Meanwhile, ${\hat{U}}_{M|G}\left(s,t\right)$ completely determines the entanglement between matter fermions and their gauge fields; the number of independent ${\hat{U}}_{M|G}\left(s,t\right)$ equals the Schmidt number of |B,(ψ,A)〉. In this quantum-gate interpretation of ${\hat{U}}_{G+M}\left(t\right)=e^{iS(\hat{B};\hat{A}\ldots ,\hat{\psi }\ldots )}$, the state-dynamics postulate $\delta S\left(\hat{B};\hat{A}\ldots ,\hat{\psi }\ldots \right)|B;A\ldots ,\psi \ldots \rangle =0$ might be equivalent to maximizing entanglement (information) with the least “gate action”.

The central point of the ICQFT is that the physical predictions are dual entanglement |B,(ψ,A)〉 in the Schmidt form, which encodes complete information on how gravity and matter are entangled and how matter fermions and their gauge fields are entangled as programmed by gravity*.* In particular, the reduced density operator $\rho_{G}^{\left\{s\right\}}$ in Eq. 8 for gravity (similarly for matter) is the physical predictions and thus must be also a physical observable (i.e., the “complete observable”, also known as Dirac’s observable) of the theory. This in turn implies that {|B,s〉} is the programming basis and $\rho_{G}^{\left\{s\right\}}$ (or, ${\hat{I}}_{\mathrm{Eu}}$ and $\hat{\Xi }$), the programming observable, must commute with all the constraints of the theory, e.g.

(15) $$\left[\rho_{G}^{\left\{s\right\}},H_{G+M}\right]=\left[\rho_{G}^{\left\{s\right\}},H_{G}+H_{M\text{-}G}\right]=0$$

for the Hamiltonian constraint. Here and hereafter all commutators are understood to act upon |B,(ψ,A)〉 because of the state-dynamics postulate (Eq. 2). As can be easily checked, $[\rho_{G}^{\left\{s\right\}},H_{M\text{-}G}]=0$, we have

(16) $$[\rho_{G}^{\left\{s\right\}},H_{G}]=0$$

as a result of Eq. 15. Thus, under the chosen particular basis for gravity, the gravity Hamiltonian $H_{G}$ is itself a physical observable. Interestingly, Eqs. 15 and 16 indicate that $\rho_{G}^{\left\{s\right\}}$ (as well as $H_{G}$) is also a quantum nondemolition observable (For detailed discussions on quantum nondemolition observables, see Ref. [42]).

The fact that $\rho_{G}^{\left\{s\right\}}$ and $H_{G}$ are physical observables of the theory ensures the consistency of the above considerations on dual dynamics. As a result of Eq. 16, |B,s〉 is an eigenstate of $H_{G}$ with eigenvalue $E_{G}\left(s\right)$; the ordering of ${\hat{U}}_{G}\left(t\right)$ and |B,s〉〈B,s| in ${\hat{U}}_{G+M}\left(t\right)$ (see Eq. 11) is thus not important. Meanwhile, it is easy to prove that in Eq. 13:

(17) $$S_{G+M}\left[s,t\right]=S_{G+M}\left[s\right]e^{-itE_{G}\left(s\right)}$$

such that the time-dependence of $S_{G+M}\left[s,t\right]$ is solely from $e^{-itE_{G}\left(s\right)}$.

To illustrate the dynamics of the trinary fields further, we can also work in Schrödinger’s picture. To this end, note first that it is meaningless to consider the time evolution of the whole system in |B,(ψ,A)〉, which is timeless and encodes complete physical information on the whole spacetime (of course the whole time) and all matter contents (if we include all matter Hamiltonians in $H_{G+M}$). However, it is still meaningful to consider the time evolution of an individual constituent state |B,s;t〉⊗|(ψ,A),s;t〉 of $|B,(\psi ,A)\rangle =\sum_{s}S_{G+M}\left[s\right]|B,s;t\rangle ⊗|(\psi ,A),s;t\rangle$, in which the time-dependence of |B,s;t〉 and |(ψ,A),s;t〉 is explicitly shown. Note that, as a result of Eq. 7:

(18) $$\left(H_{G}+H_{M\text{-}G}\right)|B,(\psi ,A)\rangle =i\frac{\partial }{\partial t}|B,(\psi ,A)\rangle =0$$

such that:

(19) $$\begin{array}{cc} & \sum_{s}S_{G+M}\left[s\right][H_{G}|B,s;t\rangle ⊗|(\psi ,A),s;t\rangle \\ & +|B,s;t\rangle ⊗H_{M|G}\left(\hat{\psi },\hat{A};s\right)|(\psi ,A),s;t\rangle ]=0 \end{array}$$

Let us define $\left|(\psi ,A),s;t\right]\equiv \langle B,s;t|B,(\psi ,A)\rangle =S_{G+M}\left[s\right]|(\psi ,A),s;t\rangle$. Note that |(ψ,A),s;t] is unnormalized and its inner product $[(\psi ,A),s;t\left|(\psi ,A),s;t\right]=S_{G+M}^{2}\left[s\right]$ represents the probability of finding the whole system in |B,s;t〉. Now if we require that

(20) $$i\frac{\partial }{\partial t}|B,s;t\rangle =H_{G}\left({\hat{B}}_{a}^{i},{\hat{E}}_{b}^{j}\right)|B,s;t\rangle$$

then using Eqs. 18 and 19 we have $i\frac{\partial }{\partial t}\left|(\psi ,A),s;t\right]=\langle B,s;t|H_{M\text{-}G}|B,(\psi ,A)\rangle$. If $S_{G+M}\left[s\right]$ is time-independent as it must be in Schrödinger’s picture, we have at once

(21) $$i\frac{\partial }{\partial t}|(\psi ,A),s;t\rangle =H_{M|G}\left(\hat{\psi },\hat{A},s;t\right)|(\psi ,A),s;t\rangle$$

in which the orthogonality of {|B,s;t〉} and {|(ψ,A),s;t〉} is used.

The dynamical equations as given in Eqs. 20 and 21 solve the problem of time in Schrödinger’s picture. The solution resembles the Page-Wootters mechanism [43] and in particular its very recent version [44]. However, in the context of quantum mechanics it is hard to associate time with a quantum degree of freedom. Fortunately, in quantum gravity we do have the desired quantum degree of freedom as spacetime itself is quantized.

Another problem related to the problem of time is how to reconcile the apparent macroscopic irreversibility (e.g., the second law of thermodynamics) with the time-symmetry of microscopic laws. This is known as the paradox of time’s arrow that has puzzled physicists at least since Boltzmann. Here, rather than reviewing the history of this long-standing problem, we give a simple argument showing that our theory might have an arrow of time. In QFT, the time symmetry is embodied by the invariance under a time-reversion operator $\hat{T}$ (${\hat{T}}_{G}$ for gravity and ${\hat{T}}_{M}$ for matter), which can be defined for every quantized field. A basic property of entanglement is that it does not decrease under any local unitary transformations (such as charge conjugation $\hat{C}$ and space inversion $\hat{P}$). If we could assume that entanglement also does not decrease under any local anti-unitary transformations like time inversion $\hat{T}$—an unproven statement to the best of the author’s knowledge, then in our case, even if the theory has time-symmetry in the usual sense, dual entanglement in the time-inversion state ${\hat{T}}_{G}{\hat{T}}_{M}|B,(\psi ,A)\rangle$ never decreases under time inversion (e.g., ${\hat{T}}_{G}$ and ${\hat{T}}_{M}$) upon the Hilbert space of each field. If this is indeed the case, our theory could have an *entanglement-induced arrow of time* [27] and allow time-asymmetry at the most fundamental level; for a further discussion supporting the above argument on this issue, see below, Section **9**. The CPT symmetry for spin-foam fermions was discussed in Ref. [46]; full consideration of the CPT symmetry in our theory will be given in future.

## 5 Conceptual aspects of information-complete description

Eqs. 20 and 21 imply that Einstein’s equation in quantum domain is separated into two pieces, one purely for spacetime and another for matter programmed by spacetime. This eliminates the Hilbert-space inconsistency of Einstein’s equation. It should be emphasized that in our picture, gravity plays a unique role as the programming system, whose Hilbert space supports information-complete field operators [27]. By contrast, either the fermion field or its gauge field alone is information-incomplete; only jointly they are information-complete physical entity. Thus, quantizing gravity alone is meaningful within current loop quantum gravity of remarkable success. On the other hand, we must put the information-completeness and the matter contents into a trinary description to complete a consistent quantum theory of gravity coupled with matter, thus paving the way to consistently quantize the matter sector as well. However, the matter sector within quantized spacetime is currently not well understood and progress has been made steadily [8], [45], [46], [47]. In this regard, it is reasonable to expect that the ICQFT will play a role in further development on quantum gravity, especially on quantization of the matter sector.

One should notice a subtle issue in the above considerations. When we use the evolution $|B,(\psi ,A)\rangle ={\hat{U}}_{G+M}\left(t\right)|\emptyset \rangle$ with a factorizable ${\hat{U}}_{G+M}\left(t\right)$ (see Eq. 11), the matter states $|(\psi ,A),s;t\rangle ={\hat{U}}_{M|G}\left(s,t\right)|\emptyset_{M}\rangle$ must span an orthonormal basis such that they are physical as well. This statement, together with Eq. 12 and the existence of the programming basis {|B,s〉} for gravity, can be regarded as the *definition of physical Hamiltonians for matter and gravity*. The usual Hamiltonians for matter and gravity are obtained from the gravity-matter action under the requirements of the invariance under local Lorentz transformations, local gauge transformations, and diffeomorphism. Are they identical to the physical Hamiltonians for matter and gravity as required by our theory? If the answer to this open question is “no”, then it is ready to see that our information-complete trinary description puts stronger and more restrictions on quantum fields than the usual formalism, as we pointed out above. The restrictions, which modify the usual Hamiltonians, stem from and are enforced by dual entanglement.

Now it is time for some remarks about the general feature of the ICQFT, which is a field-theoretic generalization of a new quantum formalism [27] developed recently. As the information-complete trinary description, the ICQFT does not require the measurement postulate and shares dual dynamics and dual entanglement structure of the trinary fields; kinematics and dynamics is indivisible, too: While all dynamical information is completely encoded in dual entanglement of spacetime and matter, kinematics about states and observables for an individual field is either meaningless or information-incomplete; only states and observables involved in dual entanglement (joint properties of the trinary fields) are of dynamical and physical significance. In this way, a huge number of unphysical degrees of freedom, while appearing in conventional QFT, is eliminated. Moreover, the ICQFT has a uniquely determined “initial condition”, namely, |∅〉, which is a physical state and means *absolute nothing and nowhere*, in sharp contrast to the concept of vacuum in conventional QFT. As spacetime and matter are mutually defined via spacetime-matter entanglement in the ICQFT, |∅〉 is a state of no matter and no spacetime and, particularly, does not correspond to a flat spacetime, which is simply empty and meaningless in our theory as there is no matter to define it.

In the information-complete trinary description under study, all matter fields and even spacetime are quantized, and as such there is simply no any room for classical entities such as meaurement apparatus and observers. Then in such a description, the theory has to consider a self-defining/explaining quantum world without appealing to any physical entity beyond the theoretic structure itself. The quantum formalism given here has the feature as stated. Let us recall that, even in loop quantum gravity (as well as in superstring theory, of course), the interpretational and conceptual problems of quantum foundations remain there—They root deeper than quantization of spacetime and call for a major conceptual step for their resolution. In other words, current quantum theory is itself an unfinished revolution, not simply because spacetime is not quantized, but rather because its own formulation calls for a radical change.

## 6 Spin-network states as programming states

In the formulation of the ICQFT given above, it is of course advantageous to have the explicit form of the programming basis {|B,s〉}. Here we give argument which supports the spin-network states spanning the programming basis. Without the input of known results for loop quantum gravity, the follow-up considerations would be too formal and of less predictive power.

For an abstract graph Γ (with nodes labeled by n and oriented links labeled by l) in three-dimensional region R with two-dimensional surface ϝ embedded, a spin-network state $|\Gamma ,j_{l},i_{n}\rangle$, where $j_{l}$ is an irreducible j representation of SU(2) for each link l and $i_{n}$ the SU(2) intertwiner for each node n, is the common eigenstates [7], [9] of the area operator $\hat{A}\left(ϝ\right)$ [with eigenvalue $A(j_{l})$ for the link l] and the volume operator $\hat{V}\left(R\right)$ [with eigenvalue $V(i_{n})$ for the node n]. Then, $|\Gamma ,J,I\rangle =|\Gamma ,j_{1}\ldots j_{L},i_{1}\ldots i_{N}\rangle$ represents a spin-network state for N quanta of volume, separated from each other by the adjacent surfaces of L quanta of area. The spin-network states, once defined in a diffeomorphism invariant way, span an orthonormal basis ($\langle \Gamma ,J^{\prime },I^{\prime }|\Gamma ,J,I\rangle =\delta_{\Gamma^{\prime }\Gamma }\delta_{J^{\prime }J}\delta_{I^{\prime }I}$) for the physical Hilbert space of quantized gravity.

On the other hand, quantum states of gravity coupled with matter in loop quantum gravity read [7], [9]:

(22) $$|\Gamma ,j_{l},i_{n}\rangle ⊗|k_{l},F_{n},w_{n}\rangle$$

where $k_{l}$ is the electric flux across the surface l and $F_{n}$ ($w_{n}$) represents the number of fermions (field strength) at node n; the Higgs field is not included and will be considered elsewhere [28]; here the number ${\bar{F}}_{n}$ of anti-fermions at node n is not included for notation simplicity. These states show explicitly the correlations between the spin-network states $|\Gamma ,j_{l},i_{n}\rangle$ and the matter states $|k_{l},F_{n},w_{n}\rangle$, similarly to the correlations between |B,s〉 and |(ψ,A),s〉 in Eq. 5. Therefore, it is a natural assumption to identify |B,s〉 with $|\Gamma ,j_{l},i_{n}\rangle$. Consequently, the spin-network states $|\Gamma ,j_{l},i_{n}\rangle$ are physical prediction, and the geometry operators $\hat{A}\left(ϝ\right)$ and $\hat{V}\left(R\right)$ defined in a diffeomorphism invariant manner [1], [9] are physical observables.

Then $H_{M\text{-}G}\left(\hat{\psi },\hat{A};{\hat{B}}_{a}^{i},{\hat{E}}_{b}^{j}\right)$ in Eq. 4 can be expanded in terms of $|\Gamma ,j_{l},i_{n}\rangle$ as

(23) $$H_{M\text{-}G}^{\left\{\Gamma \right\}}=\sum_{\begin{array}{c} l\in \Gamma \cap ϝ\\ \Gamma ,n\in R \end{array}}|\Gamma ,j_{l},i_{n}\rangle \langle \Gamma ,j_{l},i_{n}|⊗H_{M|G}^{\Gamma (j_{l},i_{n})}\left(\hat{\psi },\hat{A}\right)$$

where $H_{M|G}^{\Gamma (j_{l},i_{n})}\left(\hat{\psi },\hat{A}\right)$ is the programmed matter Hamiltonian associated with $|\Gamma ,j_{l},i_{n}\rangle$ and $H_{M\text{-}G}^{\left\{\Gamma \right\}}\equiv H_{M\text{-}G}$. In this way, the spin-network states, while defining the intrinsic geometry [1], [7], [9], are interpreted here to be correlated with the states generated by the matter Hamiltonian to ensure that spacetime and matter are mutually measured and entangled; $|\Gamma ,j_{l},i_{n}\rangle$ are then physical states (namely, the physical predictions of the theory) that represent not merely geometry without matter contents.

As is already known in loop quantum gravity, the geometry operators $\hat{A}\left(ϝ\right)$ and $\hat{V}\left(R\right)$ are “partial observables” as named by Rovelli [7]. In a conceptually clear way, Thiemann [1] argued that the geometry operators become diffeomorphism invariant and thus physical as soon as they couple with matter excitations. According to the above-mentioned general feature of the ICQFT, spacetime-matter entanglement is the deeper physics underlying Thiemann’s argument. This is the very reason why only those $|\Gamma ,j_{l},i_{n}\rangle$ appearing in spacetime-matter entanglement are physical predictions of our theory: Here the geometry quanta are counted/measured only by matter excitations and there is no counting if no matter excitations; in this sense, *physical geometry* is a joint property of spacetime *and* matter. This leads to a huge truncation of the spin-network states for gravitational Hilbert space as imposed by the state-dynamics unification of our formalism, namely, the indivisibility of kinematics and dynamics.

As $|\Gamma ,j_{l},i_{n}\rangle$ are the common eigenstates of $\hat{A}\left(ϝ\right)$ and $\hat{V}\left(R\right)$, one obviously has

(24) $$\left[\hat{A}\left(ϝ\right),H_{M\text{-}G}^{\left\{\Gamma \right\}}\right]=\left[\hat{V}\left(R\right),H_{M\text{-}G}^{\left\{\Gamma \right\}}\right]=0$$

Meanwhile, $\hat{A}\left(ϝ\right)$ and $\hat{V}\left(R\right)$ as physical observables of the theory should commute with all the constraints. In particular, we have

(25) $$\left[\hat{A}\left(ϝ\right),H_{G}+H_{M\text{-}G}^{\left\{\Gamma \right\}}\right]=\left[\hat{V}\left(R\right),H_{G}+H_{M\text{-}G}^{\left\{\Gamma \right\}}\right]=0$$

implying

(26) $$\left[\hat{A}\left(ϝ\right),H_{G}\right]=\left[\hat{V}\left(R\right),H_{G}\right]=0$$

as a result of Eq. 24. Eq. 26 implies that $|\Gamma ,j_{l},i_{n}\rangle$ are also the eigenstates of the Hamiltonian $H_{G}$ for the gravity sector, and in particular

(27) $$\left[H_{G},H_{M\text{-}G}^{\left\{\Gamma \right\}}\right]=\left[H_{G},H_{G+M}\right]=0.$$

Similarly to the above general discussions on dynamics, we can also consider the time evolution of an individual spin-network state (or any superposition of a given set of spin-network states). In this case, the spin-network states are not defined in spacetime; rather, they *are* spacetime [7]. So we can include the explicit time- and field-dependences for the spin-network states by $|B,\Gamma ,j_{l},i_{n};t\rangle$, as well as for the matter states by $|\left(\psi ,A\right),\Gamma ,k_{l},F_{n},w_{n};t\rangle$ such that the pair-equations as in (20) and (21) can be obtained. Meanwhile, in Heisenberg’s picture Eq. 5 is rewritten as

(28) $$\begin{array}{cc} |B,(\psi ,A)\rangle & =\sum_{\begin{array}{c} l\in \Gamma \cap ϝ\\ \Gamma ,n\in R \end{array}}S_{G+M}\left[\Gamma ,n,l;t\right]|B,\Gamma ,j_{l},i_{n}\rangle \\ & ⊗|\left(\psi ,A\right),\Gamma ,k_{l},F_{n},w_{n}\rangle \end{array}$$

which is generated by $|B,(\psi ,A)\rangle ={\hat{U}}_{G+M}\left(t\right)|\emptyset \rangle$ with

(29) $$\begin{array}{cc} {\hat{U}}_{G+M}\left(t\right) & =\sum_{\begin{array}{c} l\in \Gamma \cap ϝ\\ \Gamma ,n\in R \end{array}}|B,\Gamma ,j_{l},i_{n}\rangle \langle B,\Gamma ,j_{l},i_{n}|{\hat{U}}_{G}\left(t\right)\\ & ⊗{\hat{U}}_{M|G}^{\Gamma (j_{l},i_{n})}\left(k_{l},F_{n},w_{n},t\right) \end{array}$$

As we noticed previously, the time-dependence of $S_{G+M}$ comes only from a phase factor that can be removed by redefining $|B,\Gamma ,j_{l},i_{n}\rangle$.

The picture underlying Eq. 29, similar to the above quantum-gate interpretation of ${\hat{U}}_{G+M}\left(t\right)$, is physically clear and compelling: ${\hat{U}}_{G}\left(t\right)$ creates from no spacetime a superposition of the spin-network states and in the meanwhile, via programmed entanglement operations ${\hat{U}}_{M|G}^{\Gamma (j_{l},i_{n})}\left(k_{l},F_{n},w_{n},t\right)$ generates matter states from no matter, mamely

(30) $$\begin{array}{cc} {\hat{U}}_{G}\left(t\right)|\emptyset_{G}\rangle & =\sum_{\begin{array}{c} l\in \Gamma \cap ϝ\\ \Gamma ,n\in R \end{array}}S_{G+M}\left[\Gamma ,n,l;t\right]|B,\Gamma ,j_{l},i_{n}\rangle \\ {\hat{U}}_{M|G}^{\Gamma (j_{l},i_{n})}|\emptyset_{M}\rangle & =|\left(\psi ,A\right),\Gamma ,k_{l},F_{n},w_{n}\rangle \end{array}$$

Obviously, $|\left(\psi ,A\right),\Gamma ,k_{l},F_{n},w_{n}\rangle$ also defines a graph (the “matter graph”) with nodes and links. Then spacetime-matter entanglement is actually quantum correlations between the spacetime graphs and the matter graphs. What is the relation, if any, between the matter graphs and the Feynman graphs? This is certainly an interesting future issue.

Needless to say, if the spin-network states indeed span the programming basis as we assumed in this Section and the next Section, then physical picture underlying our formulation is surely more transparent; many fruitful results on, e.g., quantum geometry, are available. Regarding this, it remains to be seen that the gravitational Hamiltonian in loop quantum gravity is indeed a physical Hamiltonian. Meanwhile, the Euclidean entanglement action ${\hat{I}}_{\mathrm{Eu}}$ in this case can be given totally in terms of the physical operators $\hat{A}\left(ϝ\right)$ and $\hat{V}\left(R\right)$ for geometry; later on we will show a particular example of this situation (see Eq. 42 below).

## 7 Quantum information definition of dark energy

The explicit form of $H_{M\text{-}G}\left(\hat{\psi },\hat{A};{\hat{B}}_{a}^{i},{\hat{E}}_{b}^{j}\right)$ [1], [7], [8] shows that there are two kinds of the gravity-matter coupling terms. Two terms are *two-party couplings* (denoted by $H_{2p}^{\left\{\Gamma \right\}}$), each corresponding to the interaction between gravity and matter fermions, or between gravity and gauge fields; *there is only one three-party coupling* term (denoted by $H_{3p}^{\left\{\Gamma \right\}}$) describing the interaction of gravity, matter fermions, and gauge fields. As a result, in the Hilbert space of matter only the three-party coupling term is responsible for entanglement between matter fermions and gauge fields, as programmed by $|B,\Gamma ,j_{l},i_{n}\rangle$, while the two-party couplings do not change the programmed matter entanglement. Meanwhile, in the presence of matter, every link of the graph is labeled by the irreducible j representation of SU(2) and the irreducible representation of the gauge group, while fermions locate on the nodes. Intuitively, links of the graph are the Faraday lines of forces [7]; if there is no link, there is no interaction. This intuitive picture motivates us to rewrite $H_{M\text{-}G}^{\left\{\Gamma \right\}}=H_{2p}^{\left\{\Gamma \right\}}+H_{3p}^{\left\{\Gamma \right\}}$ as:

(31) $$\begin{array}{cc} H_{2p}^{\left\{\Gamma \right\}} & =\sum_{\Gamma ,n\in R}|\Gamma ,j_{l}=\emptyset ,i_{n}\rangle \langle \Gamma ,j_{l}=\emptyset ,i_{n}|\\ & ⊗H_{M|G}^{\Gamma (i_{n})}\left(\hat{\psi },\hat{A}\right)\\ H_{3p}^{\left\{\Gamma \right\}} & =\sum_{\begin{array}{c} l\in \Gamma \cap ϝ\\ \Gamma ,n\in R \end{array}}|\Gamma ,j_{l}\neq \emptyset ,i_{n}\rangle \langle \Gamma ,j_{l}\neq \emptyset ,i_{n}|\\ & ⊗H_{M|G}^{\Gamma (j_{l}\neq \emptyset ,i_{n})}\left(\hat{\psi },\hat{A}\right) \end{array}$$

here $H_{M|G}^{\Gamma (i_{n})}\left(\hat{\psi },\hat{A}\right)=H_{\mathrm{Fermi}|G}^{\Gamma (i_{n})}\left(\hat{\psi }\right)+H_{\mathrm{gauge}|G}^{\Gamma (i_{n})}\left(\hat{A}\right)$; $H_{\mathrm{Fermi}|G}^{\Gamma (i_{n})}$ [$H_{\mathrm{gauge}|G}^{\Gamma (i_{n})}$] is the Hamiltonian of matter fermions (gauge fields) resulting from the two-party couplings $H_{2p}^{\left\{\Gamma \right\}}$ in the Hilbert space of matter.

As the programmed matter entangled state $|\left(\psi ,A\right),\Gamma ,k_{l},F_{n},w_{n}\rangle$ is the energy eigenstate (see, e.g., Ref. [45] for simple examples of the energy eigenstates in loop quantum gravity) of $H_{M|G}^{\Gamma (j_{l},i_{n})}\left(\hat{\psi },\hat{A}\right)$, the corresponding eigenvalue should have the following form

(32) $$E_{M|G}^{\Gamma (j_{l},i_{n})}=\left\{ \begin{array}{c} {\bar{E}}_{M|G}^{\Gamma (i_{n})}\left(F_{n},w_{n}\right)\;\;\text{(without}\;\text{link}\;\text{exc.)}\\ E_{M|G}^{\Gamma (j_{l},i_{n})}\left(F_{n},w_{n};k_{l}\right)\;\;\text{(with}\;\text{link}\;\text{exc.)} \end{array}\right.$$

where the “node energy” ${\bar{E}}_{M|G}^{\Gamma (i_{n})}\left(F_{n},w_{n}\right)$ [the “link energy” $E_{M|G}^{\Gamma (j_{l},i_{n})}\left(F_{n},w_{n};k_{l}\right)$] is the energy eigenvalue of $H_{M|G}^{\Gamma (i_{n})}\left(\hat{\psi },\hat{A}\right)$ [$H_{M|G}^{\Gamma (j_{l}\neq \emptyset ,i_{n})}\left(\hat{\psi },\hat{A}\right)$] related to eigenstate $|\left(\psi ,A\right),\Gamma ,k_{l}=\emptyset ,F_{n},w_{n}\rangle$ without link excitations [$|\left(\psi ,A\right),\Gamma ,k_{l}\neq \emptyset ,F_{n},w_{n}\rangle$ with link excitations]. As noticed above, only those $|(\psi ,A),\Gamma ,k_{l}\neq \emptyset ,F_{n},w_{n}\rangle$ with link excitations have programmed matter entanglement such that matter fermions and gauge fields are mutually defined and measured. For those $|(\psi ,A),\Gamma ,k_{l}=\emptyset ,F_{n},w_{n}\rangle$ without link excitations, matter fermions and gauge fields couple merely with gravity and as such, ${\bar{E}}_{M|G}^{\Gamma (i_{n})}\left(F_{n},w_{n}\right)$ must be *dark energy*, which stems from the two-party couplings and relates only to the volume excitations. In other words, the ICQFT allows us to have a theoretical definition of dark energy to be a kind of the bulk/volume energy, while the surface/area energy related to matter links is the “visible energy”.

Based on the above observations we can put |B,(ψ,A)〉 into a superposition of a “dark-energy state” |dark〉 and a “visible-energy state” |visi〉, namely

(33) $$|B,(\psi ,A)\rangle =p_{D}|\mathrm{dark}\rangle +q_{V}|\mathrm{visi}\rangle$$

where ${\left|p_{D}\right|}^{2}+{\left|q_{V}\right|}^{2}=1$ and

(34) $$\begin{array}{cc} |\mathrm{dark}\rangle & =\sum_{\Gamma ,n\in R}S_{G+M}^{D}\left[\Gamma ,n\right]|\Gamma ,i_{n}\rangle ⊗|\Gamma ,F_{n},w_{n}\rangle \\ |\mathrm{visi}\rangle & =\sum_{\begin{array}{c} l\in \Gamma \cap ϝ\\ \Gamma ,n\in R \end{array}}S_{G+M}^{V}\left[\Gamma ,n,l\right]|\Gamma ,j_{l}\neq \emptyset ,i_{n}\rangle \\ & ⊗|\Gamma ,k_{l}\neq \emptyset ,F_{n},w_{n}\rangle \end{array}$$

By definition, 〈visi|dark〉=0. While for |dark〉 spacetime and the fermion/gauge field are mutually defined and measured, the fermion field and the gauge field are mutually defined and measured (i.e., visible to each other) for |visi〉, as programmed by gravity.

## 8 Emergence of classical Einstein equation from spacetime-matter entanglement

Is the ICQFT given above a candidate theory of quantum gravity plus matter? Here let us go further to illustrate one of the possible physical consequences implied by a particular form of spacetime-matter entanglement, hoping to offer a positive answer to this question. As the ICQFT is a concrete formalism of interacting spacetime and matter, the state |B;A…,ψ…〉 for spacetime and all matter contents contains complete physical predictions of our Universe. Yet, how to calculate explicitly the state, which generally includes infinite parameters, for physically interesting situations is a future challenge, unless we have the entanglement Hamiltonian or the Euclidean entanglement action. Before doing any explicit calculations, one can consider specific spacetime-matter entanglement that is well-based from other sides of existing quantum gravity problems.

To this end, we specify dual spacetime-matter entanglement in Eq. 5 as

(35) $$|G,M\rangle =\frac{1}{\sqrt{Z}}\sum_{s}e^{-Z_{s}/2}|G,s\rangle ⊗|M,s\rangle$$

where the Schmidt bases for the gravity and matter sectors are denoted collectively by |G,s〉 and |M,s〉, respectively. Here Z is a normalization constant and $Z_{s}$ stands for the possible spectra for the gravitational and matter states. Actually, gravity and matter are isospectral as their reduced density operators read $\rho_{G}=\frac{1}{Z}\sum_{s}e^{-Z_{s}}|G,s\rangle \langle G,s|$ and $\rho_{M}=\frac{1}{Z}\sum_{s}e^{-Z_{s}}|M,s\rangle \langle M,s|$; for applications of pure-state entanglement like that in Eq. 35 in a thermodynamic context, see [48]. Now let us suppose that |M,s〉 (|G,s〉) is an energy eigenvector of matter’s Hamiltonian $H_{M|G}$ (an area eigenvector of the area operator $\hat{A}$) with eigenvalue $E_{s}^{M}$ ($A_{s}^{G}$) such that

(36) $$\beta E_{s}^{M}=\tilde{\beta }A_{s}^{G}=Z_{s}$$

with β and $\tilde{\beta }$ being two constant factors.

If the matter field experiences a constant acceleration a, a Rindler horizon appears due to the acceleration. As a uniformly accelerated observer in Minkowski spacetime has no access to the states inside the Rindler horizon, the reduced state for matter outside the Rindler horizon is a thermal state characterized by the Unruh temperature $T_{U}=\frac{a\hbar }{2\pi c\kappa_{B}}$, where $\kappa_{B}$ and the speed of light c are explicitly included. This is known as the Unruh effect [18], [19], [20] uncovered by a semi-classical analysis without quantizing gravity. Some recent results [49], [50], [51], [52], [53], [54] studied the black-hole physics making use of the fact that the near-horizon geometry of non-extremal black holes, as seen by a stationary observer, is descriable by a local Rindler horizon. In these studies (e.g., [50], [51], [52], [53], [54]), entanglement between the inside and the outside of the Rindler horizon is associated with the black-hole entropy.

Instead of these previous results, here we consider whether or not the spacetime-matter entangled state |G,M〉 could account for the Unruh effect. For this purpose, one can identify $\beta =\frac{1}{\kappa_{B}T_{U}}$ in Eq. 36 such that $\rho_{M}$ is indeed a thermal state at the Unruh temperature $T_{U}$. When there is a small perturbation to the whole system, the reduced density operators will be $\rho_{G}^{\prime }=\rho_{G}+\delta \rho_{G}$ and $\rho_{M}^{\prime }=\rho_{M}+\delta \rho_{M}$. The change of the spacetime-matter entanglement entropy at the first-order in δρ reads

(37) $$\delta E_{\mathrm{GM}}=\tilde{\beta }\delta \langle \hat{A}\rangle \equiv \tilde{\beta }\delta A=\beta \delta \langle H_{M|G}\rangle \equiv \beta \delta E_{M|G}$$

Here we have used Eq. 36, as well as the facts (see, e.g., [51], [52]) that $\delta E_{\mathrm{GM}}=-\mathrm{tr}\left[\delta \rho_{G}\ln\rho_{G}\right]=-\mathrm{tr}\left[\delta \rho_{M}\ln\rho_{M}\right]$ and $\mathrm{tr}[\delta \rho_{M}]=0$. To be consistent with the Bekenstein-Hawking area law [55], [56], [57], one only needs to choose $\tilde{\beta }$ to be a universal constant $\tilde{\beta }=\frac{1}{4\ell_{P}^{2}}$, where the Planck length $\ell_{P}=\sqrt{G\hbar /c^{3}}$, such that

(38) $$\delta E_{\mathrm{GM}}=\frac{\delta A}{4\ell_{P}^{2}}\equiv \frac{\delta A}{A_{0}}$$

In particular, Eq. 37 implies a relation:

(39) $$\delta E_{M|G}=\frac{ac^{2}}{8\pi G}\delta A$$

which is identical in form to the Frodden-Gosh-Perez relation [49].

The celebrated work by Jacobson [34] shows that the input of the Unruh temperature and Eq. 37 gives the classical Einstein equation. This then means that our theory of quantum gravity has a correct classical limit. A similar result was obtained within the context of loop quantum gravity [52], [54]. Moreover, if |G,s〉 is, instead, an energy eigenvector of gravity’s Hamiltonian $H_{G}$ (see Section **5**), following the above arguments and Eq. 18 yields $\delta \langle H_{G}\rangle \equiv \delta E_{G}=-\frac{ac^{2}}{8\pi G}\delta A$ with the help of the energy-area relation derived in loop quantum gravity [50], [52], [54]. In this case, while one recovers the same Bekenstein-Hawking area law as in Eq. 38, the mean energy of gravity has to be identical to that of matter, but of opposite sign such that *the total mean energy of the whole gravity+matter system is exactly zero*.

While the relations in Eqs. 38 and 39 are formally identical to those previous results [49], [50], [51], [52], [53], [54], here the physical picture is dramatically different because of different entanglement involved. Moveover, note that in dual entanglement |G,M〉, the entangled states |M,s〉 for the matter part depend of course on their physical contents (i.e., matter species). However, as gravity universally couples to matter via matter’s energy-momentum tensor $T_{\mu }^{I}$, our derivation of the relations in Eqs. 38 and 39 does not make use of any details on matter species. This explains the universal independence of $\delta E_{\mathrm{GM}}$ on matter species, known as the species problem [50], [54].

## 9 A universal spacetime-matter state of the Universe

At first sight, it seems strange that Eq. 35 has no volume excitations, unlike the Universe state |B,(ψ,A)〉 in Eq. 28. This could be explained, following a beautiful argument in Rovelli’s book [7], by the fact that in the presence of a horizon, only surface excitations are responsible for the physics, especially for entropy counting. If this is indeed the case, the volume excitations and the matter excitations programmed by them should be absent or factorized away from the surface terms *in some way*.

Here we would like to ask, besides Rovelli’ argument, if there could be any other fundamental reason explaining the absence (or presence) of the volume excitations for entropy counting of the horizon. For this purpose, we can rewrite Eq. 35 as an area-matter entangled state:

(40) $${|G,M\rangle }_{\partial \Gamma }=\sum_{\Gamma ,l\in \Gamma \cap ϝ}\frac{e^{-A\left(j_{l}\right)/2A_{0}}}{\sqrt{Z_{\partial \Gamma }}}|G,\partial \Gamma ,j_{l}\rangle ⊗|M,\partial \Gamma ,k_{l}\rangle$$

Here the area states $|G,\partial \Gamma ,j_{l}\rangle$ are the eigenstates of $\hat{A}\left(ϝ\right)$ with eigenvalue $A(j_{l})$, ∂Γ means Γ’s boundary, i.e., its intersections with the surface ϝ; $|M,\partial \Gamma ,k_{l}\rangle$ stands for the matter states programmed by $|G,\partial \Gamma ,j_{l}\rangle$. Inspecting the derivation of Eq. 37, one easily sees that the entanglement-area relation (38) is universally valid for the area-matter entangled state in Eq. 40.

Note that the relation $\delta E_{\mathrm{GM}}=\frac{\delta A}{4\ell_{P}^{2}}$ can be regarded as a variational version of the holographic principle [58], [59], [60], called the variational holographic relation hereafter as it relates variations of two expectation values ($\delta E_{\mathrm{GM}}$ and δA). Reversing our reasoning that the specific form of ${|G,M\rangle }_{\partial \Gamma }$ leads to the variational holographic relation, we can take the variational holographic relation as a fundamental principle that any theory of quantum gravity has to satisfy. Then it is remarkable to see that the ICQFT, together with the variational holographic relation, *uniquely* determines the spacetime-matter entangled state as given in (40), in which the Schmidt coefficients $S_{G+M}$ can be specified. This encourages us to conjecture the following spacetime-matter state:

(41) $$\begin{array}{cc} |\mathrm{Univ}\rangle & =\sum_{\begin{array}{c} l\in \Gamma \cap ϝ\\ \Gamma ,n\in R \end{array}}\frac{e^{-V\left(i_{n}\right)/2V_{0}}\cdot e^{-A\left(j_{l}\right)/2A_{0}}}{\sqrt{Z_{\partial \Gamma }Z_{\Gamma }}}|G,\Gamma ,i_{n},j_{l}\rangle \\ & ⊗|M,\Gamma ,F_{n},w_{n},k_{l}\rangle \end{array}$$

where $Z_{\Gamma }$ is a new normalization constant and $V_{0}$ a volume constant to be determined. The Euclidean entanglement action ${\hat{I}}_{\mathrm{Eu}}^{\left(\Gamma +\partial \Gamma \right)}$ with respect to |Univ〉 is

(42) $${\hat{I}}_{\mathrm{Eu}}^{\left(\Gamma +\partial \Gamma \right)}=\frac{\hat{A}\left(ϝ\right)}{A_{0}}+\frac{\hat{V}\left(R\right)}{V_{0}}$$

which is purely geometric.

Now let us explain how we can arrive at |Univ〉. To this end, we return to the quantum-gate interpretation of ${\hat{U}}_{G+M}\left(t\right)$. By this interpretation, the dynamical evolution resulting in |Univ〉 [|B,(ψ,A)〉] is exactly the computing process of an information-complete quantum computer defined in Ref. [27] if we use $\Gamma_{T}$ (T=0,1,2,…) to label the computing steps, which actually defines discrete time. Note that $|B,\Gamma ,j_{l},i_{n}\rangle$ [$|\left(\psi ,A\right),\Gamma ,k_{l},F_{n},w_{n}\rangle$] in Eq. 28 is the energy eigenstate of $H_{G}$ [$H_{M|G}^{\Gamma (j_{l},i_{n})}$] according to the preceding Section. Γ thus labels the total energy of spacetime or matter for a given graph. The information-complete quantum computing proceeds from T=0 (the empty state |∅〉) and consumes matter of increasing energies step by step, resulting in expanded spacetime and more matter described by |Univ〉. During expanding spacetime and creating matter, the spacetime and matter graphs grow up and get more entangled. In this process spacetime and matter “borrow” energies from each other while keeping the total energy of the trinary fields exactly zero.

If spacetime-matter entanglement has a universal form shown in Eq. 41, the total entanglement entropy is the sum of entanglement entropies for nodes and for links—the additivity of volume and area entanglement entropies; in particular, the variational holographic relation is modified as

(43) $$\delta E_{\mathrm{GM}}^{\left(\Gamma +\partial \Gamma \right)}=\frac{\delta A}{A_{0}}+\frac{\delta V}{V_{0}}$$

which is also of a universal form. In other words, the information-complete quantum computing for spacetime and matter in |Univ〉 results in a monotone increasing, by a fixed and universal amount $\delta E_{\mathrm{GM}}^{\left(\Gamma +\partial \Gamma \right)}$ for each computing step ($\Gamma_{T}\to \Gamma_{T+1}$ for large enough T), of the spacetime-matter entanglement entropy. *This monotonically increasing entanglement entropy thus defines an arrow of time*. If we understand |Univ〉 in a cosmological context, such an arrow of time is something like the usual cosmological arrow of time. As we use the discrete computing step to label time, it is hard to relate such a discrete time with the time in the dynamical equation. Eq. 43 generalizes the variational holographic relation given in Eq. 38, which is approximately valid for large $V_{0}$.

Obviously, ${|G,M\rangle }_{\partial \Gamma }$ describes a Universe where matter can only entangle with quantized surface. In other words, the area-matter entangled state encodes complete information of physical predictions for the strictly holographic Universe, where the programming basis has to be switched from {$|\Gamma ,j_{l},i_{n}\rangle$} to {$|B,\partial \Gamma ,j_{l}\rangle$}. Such a truncation of the spin-network Hilbert space can be done, e.g., by taking the node (volume) degrees of freedom of spin-networks as pure gauge [61]. However, as we already noted, the node energy contributes to the dynamics of the whole trinary fields. Consequently, *our Universe is not strictly holographic due to the presence of dark energy*. Similarly to Eq. 36, we have $\beta_{L}E_{M|G}^{\Gamma (j_{l},i_{n})}+\beta_{N}{\bar{E}}_{M|G}^{\Gamma (i_{n})}=A\left(j_{l}\right)/A_{0}+V\left(i_{n}\right)/V_{0}$, where $\beta_{L}$ and $\beta_{N}$ are two constant factors related to the link energy and the node energy, respectively. Consequently

(44) $$\beta_{L}\delta E_{M|G}^{\Gamma }+\beta_{N}\delta {\bar{E}}_{M|G}^{\Gamma }=\frac{\delta A}{A_{0}}+\frac{\delta V}{V_{0}}$$

This relation generalizes the variational energy-area relation in Eq. 39.

## 10 The cosmological constant term

Now let us consider the application of the results, given in the above Section, to the problem of the cosmological constant [62]. Note that in loop quantum gravity, the Hamiltonian related to the cosmological constant term reads (after restoring G, ℏ, and c)

(45) $$H_{\Lambda }=-\frac{\hbar c\Lambda }{8\pi \ell_{P}^{2}}\int_{R}d^{3}x\sqrt{\det g}$$

where the 3-metric $g_{ab}=e_{a}^{i}e_{b}^{i}$ and $\int_{R}d^{3}x\sqrt{\det g}$ is classically the total volume of the region [1], [8]. If we assume that $H_{\Lambda }$ after quantization is contributed solely by the dark energy defined by our theory, we would have $\beta_{N}\delta {\bar{E}}_{M|G}^{\Gamma }=\beta_{N}\frac{\hbar c\Lambda }{8\pi \ell_{P}^{2}}\delta V=\delta V/V_{0}$ (Note that the mean value of $H_{\Lambda }$ should be identical to the dark energy, but of opposite sign). This allows us to determine $V_{0}$ as $V_{0}^{-1}=\beta_{N}\frac{\hbar c\Lambda }{8\pi \ell_{P}^{2}}$, keeping $\beta_{N}$ undetermined. If |Univ〉 describes an expanding Universe, which expands at a constant acceleration $a_{E}$, a natural conjecture might be $\beta_{N}^{-1}=\frac{\hbar a_{E}}{2\pi c}\equiv \frac{\hbar }{2\pi t_{E}}\equiv \kappa_{B}T_{E}$ such that

(46) $$V_{0}=\frac{4\ell_{P}^{2}}{ct_{E}\Lambda }$$

$V_{0}$ can indeed be very large if $\frac{1}{ct_{E}\Lambda }$ is a length scale comparable to the Hubble length $∼\sqrt{1/\Lambda }$. Thus, for the expanding Universe described by |Univ〉, the cosmological constant term in Einstein’s equation could be attributed to the dark/node energy, related to the volume quanta, defined by our theory.

Recall the thermodynamic relation dE=TdS−PdV relating energy E, entropy S, temperature T, pressure P, and volume V. For this relation to hold in the present case, we need to require $\beta_{L}=\beta_{N}$, which allows us to define the total “entanglement energy” $\Xi_{\Gamma }\equiv E_{M|G}^{\Gamma }+{\bar{E}}_{M|G}^{\Gamma }$ (see the definition of the entanglement Hamiltonian in Eq. 9). From Eq. 44 we then obtain quite similarly a thermodynamic relation:

(47) $$\delta \Xi_{\Gamma }=T_{E}\delta S-P_{U}\delta V$$

provided that

(48) $$\begin{array}{cc} \delta S & =\frac{\kappa_{B}\delta A}{A_{0}},\\ P_{U} & =-\frac{\kappa_{B}T_{E}}{V_{0}}=-\frac{\hbar c\Lambda }{8\pi \ell_{P}^{2}}=-\frac{c^{4}\Lambda }{8\pi G} \end{array}$$

It is ready to see that the extra term $\delta V/V_{0}$ in Eq. 44 gives a negative pressure $P_{U}$, which is a universal constant given by three fundamental constants c, Λ, and G. The universal negative pressure is believed to expand, at the constant acceleration $a_{E}$, our Universe. This picture is consistent with current understanding of the standard cosmology and here stems directly from our theory structure. The physical significance of Eq. 48 is transparent: *The total entanglement energy* $\Xi_{\Gamma }$ *is the physical energy*, which consists of two parts—the visible/holographic energy related to the variational holographic relation $T_{E}\delta S=\frac{\kappa_{B}T_{E}\delta A}{A_{0}}$ (see Eq. 39) and the dark energy related to $\left|P_{U}\right|\delta V$. Note that $\sqrt[{3}]{V_{0}}$ define a crossover length scale in between $\ell_{P}$ and $\sqrt{1/\Lambda }$ for the holographic and dark energies.

In Jacobson’s thermodynamic argument to derive the classical Einstein equation [34], the cosmological constant still remains to be a free parameter. If we make use of Eq. 43, rather than Eq. 37, in such an argument, our above discussion on the universal relation between entanglement entropy and geometry (area and volume) has actually fixed the cosmological constant term in Einstein’s equation. Therefore, we readily see that the spacetime-matter entangled state |Univ〉 in Eq. 41 provides more complete information of our Universe than ${|G,M\rangle }_{\partial \Gamma }$ in Eq. 40.

The conceptual application of the above results is profound. What we have done in this and the above two Sections is to consider two particular forms of spacetime-matter entangled states (${|G,M\rangle }_{\partial \Gamma }$ and |Univ〉) and their physical consequences. While ${|G,M\rangle }_{\partial \Gamma }$ is consistent with the variational holographic relation, |Univ〉 results in a more realistic relation between entanglement entropy and geometry. It is |Univ〉 that allows us to determine the cosmological constant term in Einstein’s equation. Reversing the reasoning supports |Univ〉 as a reliable quantum state of the Universe and moreover, the fact that our Universe is not strictly holographic.

## 11 Quantum state of a Schwarzschild black hole

Let us present one more application of the dual entanglement structure of our theory. We consider the limit on the information content of a spacetime region R associated with a surface ∂R of area A. Let us identify the spacetime region (elementary fermions and gauge fields associated within the spacetime region) as the P (SA) system. Then the P−SA measurability and the programmed measurability $\mathrm{SA}|{}_{P}$ defined in the ICQT [27] demand that $D_{A}=D_{S}=D$ and *maximally* $D_{P}=D^{2}$, i.e., the dimensions (denoted by $D_{A,S,P}$) of the three systems are all limited and related. These facts immediately lead to an obvious relation:

(49) $$E_{P(\mathrm{SA})}\leq \ln D_{P}$$

namely, spacetime-matter (P−SA) entanglement, as quantified by $E_{P(\mathrm{SA})}$ (the entropy of P or SA), is limited in our picture. Here, the equality applies only to the case of maximal P−SA entanglement.

Remarkably, loop quantum gravity can give a complete spectrum of the area operator, see Refs. [1], [7], [9]). Using the area spectrum and microstate counting [7] applied to a Schwarzschild black hole of surface area A, Eq. 49 becomes

(50) $$E_{P(\mathrm{SA})}\leq \ln D_{P}=\frac{A}{4\ell_{P}^{2}}$$

This is exactly what the holographic principle [58], [59], [60] implying that the information content is limited merely by the surface. In the context of the ICQFT, the holographic principle arises as a direct consequence of area-matter entanglement. From such a strong and universal limit on the allowed states of the trinary system as imposed by the ICQFT, it is ready to see, once again, that the restriction on the description of the trinary fields imposed by the information-completeness is much stronger than our current field-theoretical description.

Now let us apply the above argument to the Schwarzschild black hole of surface area A. As is now widely accepted, the black hole saturates [17] the entanglement bound in Eq. 50. This single fact is so special that it is enough for us to infer the global state of the black hole, namely, the black hole must be a *maximally information-complete* quantum system with the maximal area-matter entanglement:

(51) $${|\mathrm{BH},P(\mathrm{SA})\rangle }_{\partial \Gamma }=\frac{1}{\sqrt{D_{P}^{\left(L\right)}}}\sum_{\Gamma ,l\in \Gamma \cap \partial R}|P,\partial \Gamma ,j_{l}\rangle ⊗|\mathrm{SA},\partial \Gamma ,k_{l}\rangle \;$$

when only the surface of the black hole is concerned. Here $D_{P}^{\left(L\right)}=2^{A/4\ell_{P}^{2}}$ (see, e.g., Ref. [7]) is the dimensions of the link states $|P,\partial \Gamma ,j_{l}\rangle$; all $D_{P}^{\left(L\right)}$ matter link states $|\mathrm{SA},\partial \Gamma ,k_{l}\rangle$ are also maximally entangled and span an orthonormal basis in the matter sector. In this case the entanglement entropy of the black hole is $E_{P(\mathrm{SA})}^{\mathrm{BH},\partial \Gamma }=-\mathrm{tr}\left[\rho_{P}^{\left\{l\right\}}\ln\rho_{P}^{\left\{l\right\}}\right]=\ln D_{P}^{\left(L\right)}=\frac{A}{4\ell_{P}^{2}}$, where $\rho_{P}^{\left\{l\right\}}={\mathrm{tr}}_{\mathrm{SA}}\left({|\mathrm{BH},P(\mathrm{SA})\rangle }_{\partial \Gamma }\langle \mathrm{BH},P(\mathrm{SA})|\right)=\frac{1}{D_{P}^{\left(L\right)}}\sum_{\Gamma ,l\in \Gamma \cap \partial R}|P,\partial \Gamma ,j_{l}\rangle \langle P,\partial \Gamma ,j_{l}|$; see, as a comparison, Eq. 40, in which the area-matter state is not maximally entangled and leads to the entanglement entropy less than $\ln D_{P}^{\left(L\right)}$.

However, as we emphasized above, *the variational holographic relation is violated by our Universe* and has to be modified into the form given by Eq. 43. Thus, we must consider ∂R and R to give the global state of the black hole. As such, $D_{P}$ in Eq. 49 is the total dimensions of the spacetime state related to ∂R and R, and the black hole should be described by the total quantum state that is maximally information-complete in both volume and surface degrees of freedom, namely

(52) $$\begin{array}{cc} {|\mathrm{BH},P(\mathrm{SA})\rangle }_{\Gamma } & =\frac{1}{\sqrt{D_{P}^{\left(L\right)}D_{P}^{\left(N\right)}}}\sum_{\begin{array}{c} l\in \Gamma \cap \partial R\\ \Gamma ,n\in R \end{array}}|P,\Gamma ,i_{n},j_{l}\rangle \\ & ⊗|\mathrm{SA},\Gamma ,F_{n},w_{n},k_{l}\rangle \end{array}$$

where $D_{P}^{\left(N\right)}$ is the dimensions of the node states. Further consideration of entanglement entropy with respect to ${|\mathrm{BH},P(\mathrm{SA})\rangle }_{\Gamma }$ will be given elsewhere.

Maximal entanglement has an intriguing property called “monogamy” [63]: If two parties are maximally entangled, then they cannot be entangled with any third party. Let us discuss a possible application of this “non-shareability” of maximal entanglement in the present context. As we inferred, the black hole is maximally entangled in dual form. Then the monogamy of maximal entanglement implies that there is no way of extracting any information, via interactions, from the black hole. Namely, the black hole is “information-black”. As such, dynamical evolution of the black hole will be in some sense “frozen” from the trinary fields, namely, it is an “*entanglement death*” of matter and spacetime. However, the presence of the black hole in spacetime is detectable as it *defines* spacetime and can also absorb matter to grow up its entanglement. Such a picture on black holes seems to be in accordance with our intuition on what *is* a black hole, especially in the framework of ICQFT. However, it is quite different from our current understanding [1], [55], [56], [57] based on classical general relativity, thermodynamic argument, and QFT in curved classical spacetime.

Note that in ${|\mathrm{BH},P(\mathrm{SA})\rangle }_{\Gamma }$, there are both the volume and surface quanta (as well as the programmed matter) inside the Schwarzschild black hole. In this case, the interior of the black hole must be factorized away from quantum states for degrees of freedom outside the black hole as ${|\mathrm{BH},P(\mathrm{SA})\rangle }_{\Gamma }$ is maximally dual-entangled—external matter fields can entangle only with surface excitations near but outside the horizon, in a state approximately given by ${|G,M\rangle }_{\partial \Gamma }$ as typically $V_{0}$ is much larger than the black-hole volume. This gives a physical explanation validating the argument on the derivation of the variational holographic relation without the volume contribution in the presence of a horizon. Moreover, as ${|\mathrm{BH},P(\mathrm{SA})\rangle }_{\Gamma }$ is maximally information-complete, but regular, for the Schwarzschild black hole the singularity problem disappears for the inferred state ${|\mathrm{BH},P(\mathrm{SA})\rangle }_{\Gamma }$. Such a maximal entanglement possessed by the Schwarzschild black hole might be an ideal “resource” for quantum information processing with matter and spacetime.

What about the black-hole information paradox in the ICQFT? The successful account of the Bekenstein-Hawking entropy (as well as the cosmological constant term) totally in terms of spacetime-matter entanglement convinces us the elimination of this paradox within the ICQFT. Here, matter’s states seem to be thermal not because some modes of matter fields are thrown into a black hole which has “no hair” and thus destroys information about collapsing matter. Rather, all information of the whole system is coherently kept in dual entanglement and the thermality of matter’s states stems from an information-incomplete description, i.e., artificially tracing out the spacetime degrees of freedom within dual entanglement—The black hole as an information-complete system is not thermal; it thus does not evaporate and never destroys information. Nowhere is a non-unitary evolution allowed in the ICQFT*.* Recall that Hawking’s radiation was derived from quantum field theory in classical curved spacetime. By contrast, in the unitary and information-complete description of nature given in this work, there is simply no Hawking radiation and information is definitely conservative so that there is no room for any information loss. In essence, the black-hole information paradox originates from the information-incompleteness of current quantum description. As a comparison, loop quantum gravity helps to solve the singularity problem, but the information-loss problem of black holes becomes worse [64].

## 12 Summary and outlook

To summarize, we have introduced, very briefly as a start, the ICQFT, as quantum entanglement dynamics of spacetime and matter, which describes elementary matter fermions, their gauge fields, and gravity as an indivisible trinity, hoping to provide a coherent picture of unifying spacetime and matter. The fact that this is indeed possible could be regarded as a support on our previous argument on the information-complete quantum theory. Complete information of the trinary fields is encoded in the dual entanglement—spacetime-matter entanglement and matter-matter (matter fermions and their gauge fields) entanglement. Thus, in terms of entanglement, both spacetime and matter are unified as information. Here, entanglement is universal just like that gravity is universal; the universal entanglement glues spacetime and matter and is thus the building block of the world. Working in dual entanglement formalism of the trinary fields has an obvious advantage in that all constraint conditions are automatically solved and all predictions of the theory are exactly physical (neither more nor less) and explicit. We give a consistent framework of the dual dynamical evolution of the trinary fields, which accounts for the two pieces of Einstein’s equation.

Any reliable theory of quantum gravity must, first of all, make progress on existing conceptual problems, which remain to be “a major obstacle for the final construction of a quantum theory of gravity and its application to cosmology”[36]. As a concrete formulation of quantum theory for spacetime and matter, several conceptual progresses, we believe, have been made within our theory as follows.

**No probability and no observer** Our information-complete quantum description does not rely in any way on the concepts of probability and observer, or any related classical concepts in its own formulation—A probability description appears only as an information-incomplete and approximate description of nature; our Universe is self-defining and self-explaining via its trinary constituents, but not defined and explained via any external observers, as shown previously in a new quantum structure beyond current quantum theory [27]. This eliminates the conceptual obstacle of applying conventional quantum theory to cosmology. The conjectured quantum state of the Universe leads to encouraging results.

**Dark energy** The ICQFT provides a quantum information definition of the mysterious dark energy, which stems from the two-party couplings within the spacetime-matter Hamiltonian $H_{M\text{-}G}$. For such couplings, matter fermions and their gauge fields interact merely with gravity and the related energy is thus dark energy, shown to be a kind of the node/volume energy.

**Problem of time and time’s arrow** While the overall quantum state of spacetime and matter is timeless, its particular entanglement structure allows us to define separately time evolutions for spacetime and for matter. Related to this and the basic property of entanglement, our theory implies an entanglement-induced arrow of time. The Universe, as an information-complete quantum computer, has a monotonically increasing entanglement entropy, defining also an arrow of time.

**The variational holographic relation** It is actually violated by our Universe*.* Based on a particular form of area-matter entanglement, we derived the variational holographic relation (i.e., the variational form of the entropy-area law), which can lead to the classical Einstein equation (Jacobson’s thermodynamic argument), but leaving the cosmological constant as a free parameter. A more general form of spacetime-matter entanglement results in the universal relation between entanglement entropy and geometry (see Eq. 43), which modifies the variational holographic relation]. Therein, the extra term ($\delta V/V_{0}$) was argued to be responsible for the cosmological constant term in Einstein’s equation. This latter fact also confirms the above-mentioned picture of dark energy as a kind of the volume energy. In a thermodynamic argument, dark energy corresponds to $\left|P_{U}\right|V$, where $P_{U}=-\frac{c^{4}\Lambda }{8\pi G}$ is a universal negative pressure expanding our Universe with the volume V.

**Quantum black hole** For a Schwarzschild black hole, we infer that it is the maximally information-complete quantum system with maximal dual entanglement, whose monogamy enables a conceptually clear understanding of the black hole. As there is no room for non-unitary evolution, there is no information-loss paradox in our information-complete trinary description of nature.

We would like to emphasize that, as usual quantum mechanics, current QFT is also information-incomplete and describes elementary fermion fields or/and gauge fields as isolated, physical entities. This description leads to interpretational difficulties such as the black-hole information paradox and physical meaning of field quanta in curved spacetime. In the ICQFT, however, a dramatically different picture arises. Here spacetime (gravity) and matter are mutually defined and entangled—no spacetime implies no matter, and vice versa. As programmed by spacetime, elementary fermion fields and their gauge fields are likewise mutually defined and entangled; either of them alone cannot be information-complete. In some sense, it is the quantum version of Einstein’s gravity that completes the picture. The ICQFT, free of those interpretational difficulties or paradox that we encountered in conventional QFT, calls for a radical change of our current understanding on spacetime, matter, information and reality, as well as their relations. In the ICQFT, which deals with a self-explaining Universe, spacetime and matter are unified into information (entanglement) of direct physical reality. Here, it is not the constituent parts (elementary matter fermions and gauge fields, as well as spacetime) of the Universe, but rather their relations (i.e., entanglement) that are physical*.*

We have shown thus far that for both quantum mechanical systems and quantum fields, information-complete description of trinary systems shares common features such as dual entanglement, dual dynamics, and exclusion of any classical concepts like probability description. The mere possibility of achieving this is itself a surprise and conceptually appealing. Compared to current quantum theory and general relativity, dual dynamics pertaining to dual entanglement of the trinary description is a new feature. Rather than Wheeler’s famous coinage on general relativity—“Space tells matter how to move and matter tells space how to curve” [65], here we would like to say that spacetime-matter entanglement moves matter and curves spacetime quantum mechanically; even more, it defines spacetime and matter. These claims stem from the fact that spacetime-matter entanglement leads to a correct classical limit, namely, Einstein’s equation.

Like existing approaches to quantum gravity, there are too many open questions in the framework of the ICQFT, including more physical consequences implied by the information complete trinary description, the relation between the ICQFT and conventional QFT, and so on. If this work serves as a start to stimulate someone to take into account seriously and to work out more consequences of our information-complete trinary description of nature, it is exactly the author’s hope.

## Declaration of competing interest

The authors declare that they have no conflicts of interest in this work.
